# Comprehensive evidence implies a higher social cost of CO_2_

**DOI:** 10.1038/s41586-022-05224-9

**Published:** 2022-09-01

**Authors:** Kevin Rennert, Frank Errickson, Brian C. Prest, Lisa Rennels, Richard G. Newell, William Pizer, Cora Kingdon, Jordan Wingenroth, Roger Cooke, Bryan Parthum, David Smith, Kevin Cromar, Delavane Diaz, Frances C. Moore, Ulrich K. Müller, Richard J. Plevin, Adrian E. Raftery, Hana Ševčíková, Hannah Sheets, James H. Stock, Tammy Tan, Mark Watson, Tony E. Wong, David Anthoff

**Affiliations:** 1 Resources for the Future, Washington, DC USA; 2 School of Public and International Affairs, Princeton University, Princeton, NJ USA; 3 Energy and Resources Group, University of California, Berkeley, CA USA; 4 Environmental Protection Agency, Washington, DC USA; 5 Marron Institute of Urban Management, New York University, Brooklyn, NY USA; 6 NYU Grossman School of Medicine, New York, NY USA; 7 EPRI, Palo Alto, CA USA; 8 Department of Environmental Science and Policy, University of California, Davis, CA USA; 9 Department of Economics, Princeton University, Princeton, NJ USA; 10 Independent researcher, Portland, OR USA; 11 Departments of Statistics and Sociology, University of Washington, Seattle, WA USA; 12 Center for Statistics and the Social Sciences, University of Washington, Seattle, WA USA; 13 School of Mathematical Sciences, Rochester Institute of Technology, Rochester, NY USA; 14 Department of Economics, Harvard University, Cambridge, MA USA

**Keywords:** Climate-change impacts, Environmental impact, Climate-change impacts, Environmental economics, Environmental impact

## Abstract

The social cost of carbon dioxide (SC-CO_2_) measures the monetized value of the damages to society caused by an incremental metric tonne of CO_2_ emissions and is a key metric informing climate policy. Used by governments and other decision-makers in benefit–cost analysis for over a decade, SC-CO_2_ estimates draw on climate science, economics, demography and other disciplines. However, a 2017 report by the US National Academies of Sciences, Engineering, and Medicine^1^ (NASEM) highlighted that current SC-CO_2_ estimates no longer reflect the latest research. The report provided a series of recommendations for improving the scientific basis, transparency and uncertainty characterization of SC-CO_2_ estimates. Here we show that improved probabilistic socioeconomic projections, climate models, damage functions, and discounting methods that collectively reflect theoretically consistent valuation of risk, substantially increase estimates of the SC-CO_2_. Our preferred mean SC-CO_2_ estimate is $185 per tonne of CO_2_ ($44–$413 per tCO_2_: 5%–95% range, 2020 US dollars) at a near-term risk-free discount rate of 2%, a value 3.6 times higher than the US government’s current value of $51 per tCO_2_. Our estimates incorporate updated scientific understanding throughout all components of SC-CO_2_ estimation in the new open-source Greenhouse Gas Impact Value Estimator (GIVE) model, in a manner fully responsive to the near-term NASEM recommendations. Our higher SC-CO_2_ values, compared with estimates currently used in policy evaluation, substantially increase the estimated benefits of greenhouse gas mitigation and thereby increase the expected net benefits of more stringent climate policies.

## Abstract (web summary)

Coupling advances in socioeconomic projections, climate models, damage functions and discounting methods yields an estimate of the social cost of carbon of US$185 per tonne of CO_2_—triple the widely used value published by the US government.

## Main

Policies to mitigate greenhouse gas emissions are often evaluated in terms of their net benefits to society. The net benefit of a climate policy is the difference between the economic cost of the emission reduction (the mitigation costs), and the value of the damages that are prevented by that emission reduction (climate benefits, among others). In regulatory impact analysis the climate benefits of CO_2_ emission reductions are typically computed by multiplying the change in CO_2_ emissions caused by the policy with an estimate of the SC-CO_2_. This makes the SC-CO_2_ a highly influential metric, informing analysis of a wide range of climate policies worldwide.

For more than a decade, the US government has used the SC-CO_2_ to measure the benefits of reducing carbon dioxide emissions in its required regulatory analysis of more than 60 finalized, economically significant regulations, including standards for appliance energy efficiency and vehicle and power plant emissions^2^. In the USA, the SC-CO_2_ has also been used as the basis for federal tax credits for carbon capture and storage; proposed federal carbon tax legislation; state-level zero-emission credit payments for nuclear generators and power sector planning; among other applications^3^. The SC-CO_2_ also supports decision-making by government environmental agencies in other countries (for example, Germany, Canada and Mexico), and is used in standardized corporate environmental and sustainability accounting^4^.

The SC-CO_2_ is estimated using integrated assessment models (IAMs) that couple simplified representations of the climate system and global economy to estimate the economic effects of an incremental pulse of CO_2_ emissions. These models generally follow a four-step process in which (1) projections of population and gross domestic product (GDP) inform a CO_2_ emissions pathway; (2) the CO_2_ emissions path drives a climate model that projects atmospheric greenhouse gas concentrations, temperature changes and other physical variables such as sea level rise; (3) the resulting climate change impacts are monetized and aggregated as economic damages; and (4) economic discounting combines all future damages into a single present value.

In 2017, a NASEM report assessing the SC-CO_2_ estimation methodology used by the US federal government found that the leading IAMs used for estimating the SC-CO_2_ have not kept pace with recent advances in climate, economic and demographic science^1^. The NASEM report offered near-term recommendations for improving each step of the SC-CO_2_ estimation process to improve the scientific basis, characterization of uncertainty, and transparency of the SC-CO_2_. Recently, Executive Order 13990 re-established the US Interagency Working Group on the Social Cost of Greenhouse Gases (IWG) to update the federal government’s official SC-CO_2_ estimates, and to consider these NASEM recommendations in the process. Others have also criticized the models supporting the past federal SC-CO_2_ estimates for problems including damages representations that do not reflect recent science, outdated climate system models, and imperfect characterization of the compounding uncertainties affecting SC-CO_2_ estimates^5–7^.

Here we provide probabilistic SC-CO_2_ estimates from the Greenhouse Gas Impact Value Estimator (GIVE), a newly created integrated assessment model designed for quantifying the benefits of emission reductions. The model is built on the Mimi.jl platform, an open-source package for constructing modular integrated assessment models^8^. By using novel components for each step of the SC-CO_2_ estimation process, GIVE incorporates recent scientific advances that are unaccounted for by the previous generation of IAMs used in regulatory analysis. Crucially, GIVE quantifies uncertainties in each component and propagates these compounding uncertainties through the entire computation, thus allowing for a theoretically consistent valuation of the risk associated with a marginal emission of CO_2_.

Each individual component in GIVE is based on recent peer-reviewed research on socioeconomic projections, climate modelling, climate impact assessments and economic discounting. We implement GIVE with a set of internally consistent, probabilistic projections of population^9^, per capita economic growth^3,10^, and CO_2_, CH_4_ and N_2_O emissions^3^ generated using a combination of statistical modelling and expert elicitation, collectively referred to as the Resources for the Future Socioeconomic Projections^3^ (RFF-SPs). Many existing IAMs use outdated climate models and have been shown to produce temperature dynamics inconsistent with more sophisticated Earth system models^1,11^. Further, damage functions supporting previous SC-CO_2_ estimates are, to a large extent, based on studies from several decades ago^1^. A vast literature since then has expanded and improved our scientific understanding of how changes in climate are likely to affect human wellbeing^12^. To address these shortcomings, we combine socioeconomic uncertainty with probabilistic models for the climate system and damage functions (defined as functions that relate changes in climate outcomes such as temperature to economic impacts in dollars). The GIVE model employs the FaIR v1.6.2 climate model^13,14^, the BRICK sea-level model^15–17^, and updated damage function components representing the latest empirical research for the impacts of climate on agriculture^18^, mortality^19^, energy consumption^20^ and sea-level rise^21^.

Recent important contributions to the SC-CO_2_ literature have generated improvements to various components used by IAMs^22–27^ (see Supplementary Information section SI.3 for an overview of this literature). The GIVE model’s key contribution to this literature is the holistic implementation of recent advances in probabilistic socioeconomics accounting for policy uncertainty, fully quantified scientific uncertainty including climate tail risk and sea-level rise, addition of non-market sectoral damages (that is, costs not included in GDP accounting, such as mortality risk), and economic discounting tied to uncertain economic growth. These advances enable a full valuation of the risk resulting from those compounding uncertainties on the basis of improved scientific, economic and demographic evidence^3^, which have previously been unavailable. The GIVE model’s implementation of this comprehensive set of scientific improvements affirms a key result from recent work on the SC-CO_2_^22–27^, namely that improved scientific understanding of the components of SC-CO_2_ calculation leads to a higher SC-CO_2_ than has been previously used in US policymaking; moreover, our approach demonstrates this using a more robust methodology that reflects the current state of the literature. GIVE’s inputs and outputs are spatially resolved at the level of 184 countries for population, income and damages (except for agriculture damage outputs, which are resolved at 16 regions). Climate change has the potential to exacerbate existing economic inequities^6,28,29^, and our work would allow future consideration of this issue through equity weighting^30^.

We calculate the SC-CO_2_ as the discounted sum of additional damages per incremental tonne of CO_2_ produced by an emissions pulse in 2020 along an uncertain emissions trajectory derived via formal expert elicitation that reflects continued technology and policy evolution. We use an empirically calibrated stochastic discounting framework consistent with the observed behaviour of interest rates and economic growth^31^. We provide 10,000 SC-CO_2_ values using a Monte Carlo approach that samples interrelated socioeconomic, climate, and damage function uncertainties (Extended Data Table 2). The GIVE model can also be used to compute the social cost of other greenhouse gases (for example, CH_4_, N_2_O and hydrofluorocarbons).

We illustrate the relative importance of our updated model components by comparing them to outputs from the well known DICE model^32^. We also assess the sensitivity of our SC-CO_2_ estimates to our choice of sectoral, regionally disaggregated damage functions by comparing them to two aggregate, global damage functions based on meta-analyses of the broader damages literature^32,33^.

Socioeconomic projections of economic growth, population and greenhouse gas emissions represent important sources of uncertainty in the SC-CO_2_. In previous models, this uncertainty has been poorly characterized^1,34,35^. Population and growth scenarios based upon the Shared Socioeconomic Pathway (SSP)^36^ narratives, which were prominently featured in the Intergovernmental Panel on Climate Change (IPCC) Sixth Assessment Report (AR6)^14^, do not typically come with associated probabilities, though there have been efforts to assign such probabilities a posteriori on the basis of expert surveys^37^. The small number of SSPs precludes sampling the large and continuous space of possibilities that characterizes future socioeconomics and emissions. A strength of scenario-based analysis is in the qualitative exploration of uncertainty, for example through the use of bounding scenarios, including scenarios accounting for outcomes well outside the range of historical experience that become increasingly possible over very long time horizons. Such an approach does not, however, facilitate the quantitative evaluation of uncertainty and the calculation of expected values, a common requirement for policy analysis. In some cases, a lack of quantification of relative probabilities can lead to disagreements over what scenarios constitute a plausible reference case^38–40^. A holistic, probabilistic approach to accounting for these uncertainties was recently introduced^41,42^. Building on this approach, we sample the RFF-SPs, comprising multi-century probabilistic projections of population^9^ and GDP per capita^10^ at the country level as well as a distribution of projections of global CO_2_, CH_4_ and N_2_O emissions derived from a combination of statistical and expert-based approaches.

**Fig. 1:**
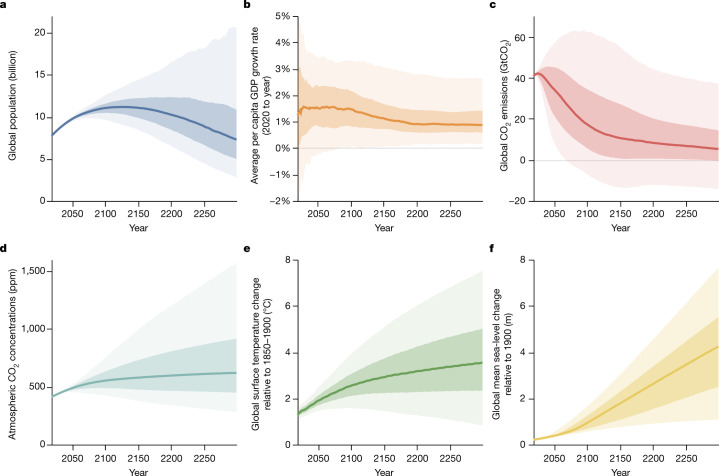
RFF-SP socioeconomic scenarios and the resulting climate system projections. **a**–**c**, Probabilistic socioeconomic projections for global population (**a**), per capita GDP growth rates (**b**), and carbon dioxide emission levels (**c**) from the RFF-SP scenarios. **d**–**f**, Corresponding climate system projections that account for parametric uncertainty in FaIR and BRICK for atmospheric carbon dioxide concentrations (**d**), global surface temperature changes relative to the 1850–1900 mean (**e**), and global mean sea-level changes relative to 1900 (**f**). In all panels, solid centre lines depict the median outcome, with darker shading spanning the 25%–75% quantile range and lighter shading spanning the 5%–95% quantile range.

The RFF-SPs complement the scenario-based approach by providing an alternative approach that characterizes the joint uncertainty across annual GDP, population and greenhouse gas emissions for the multi-century timespan required for climate damage estimation. They also leverage expert knowledge to account for potential future changes in policy and technology. The RFF-SPs project that (Fig. 1): median world population peaks at 11 billion around 2130 and subsequently declines to 7.3 billion in 2300, (2.8 billion–21 billion: 5%–95% range); median global per capita annualized economic growth declines slowly to reach a cumulative time-average rate of 0.88% between 2020 and 2300 (0.17%–2.7%: 5%–95% range); median net global CO_2_ emissions decline to 17 GtCO_2_ in 2100, which is roughly 40% of today’s levels (−7 GtCO_2_ to 62 GtCO_2_: 5%–95% range), with slower declines thereafter (see Supplementary Information section SI.1 for more detail on the RFF-SPs).

**Fig. 2:**
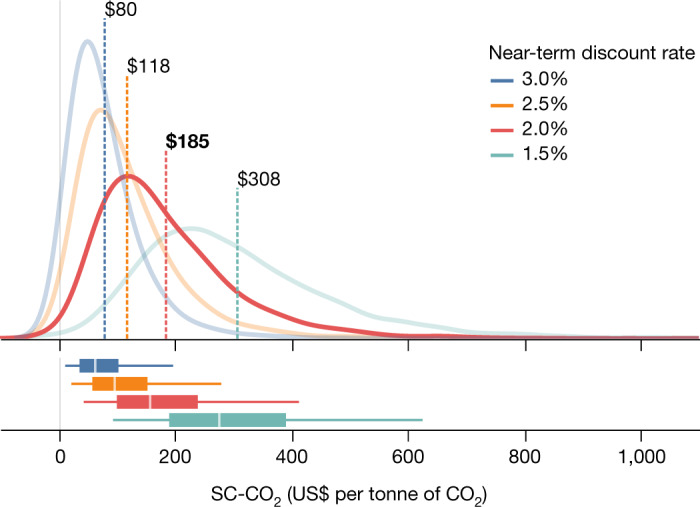
SC-CO_2_ distributions vary with the choice of near-term discount rates. Distributions of the SC-CO_2_ based on RFF-SP scenario samples, a stochastic, growth-linked discounting framework, uncertainty in the FaIR climate and BRICK sea-level models, and uncertainty in climate damage parameters. Colours correspond to near-term average discount rates of 3.0% (blue), 2.5% (orange), 2.0% (red, our preferred specification) and 1.5% (teal). Dashed vertical lines highlight mean SC-CO_2_ values. Box and whisker plots along the bottom of the figure depict the median of each SC-CO_2_ distribution (centre white line), 25%–75% quantile range (box width), and 5%–95% quantile range (coloured horizontal lines) values. All SC-CO_2_ values are expressed in 2020 US dollars per metric tonne of CO_2_.

Our mean SC-CO_2_ estimate using the preferred discounting scheme is $185 per tCO_2_ ($44–$413 per tCO_2_: 5%–95% range, in 2020 US dollars, as are all dollar results in this study) (Fig. 2). This is 3.6 times greater than the US government’s current, most commonly cited mean value of $51 per tCO_2_ using a 3% constant discount rate^43^. We report mean SC-CO_2_ values throughout this paper to align our results with the standard expected net benefit framework that is routinely used for policy analysis^44^ and supported by standard economic theory^45,46^.

SC-CO_2_ estimates are well known to be highly sensitive to the discount rate^32^ because the long residence time of CO_2_ in the atmosphere means a CO_2_ emissions pulse continues to cause damages long after it was emitted. Our preferred discounting scheme uses a 2% near-term risk-free discount rate, which reflects the recent literature on real interest rates^47–49^, which have declined substantially over recent decades^50,51^, as well as the central tendency from a survey of academic economists^52^. Our discount rate is related to stochastic consumption growth in a Ramsey-like equation, which is the commonly used approach to value marginal impacts amid uncertainty in future payoffs and consumption levels^53,54^. In this way, the parameterization of the discount rate captures risk preferences using the risk aversion parameters discussed in Methods.

**Extended Data Fig. 1:**
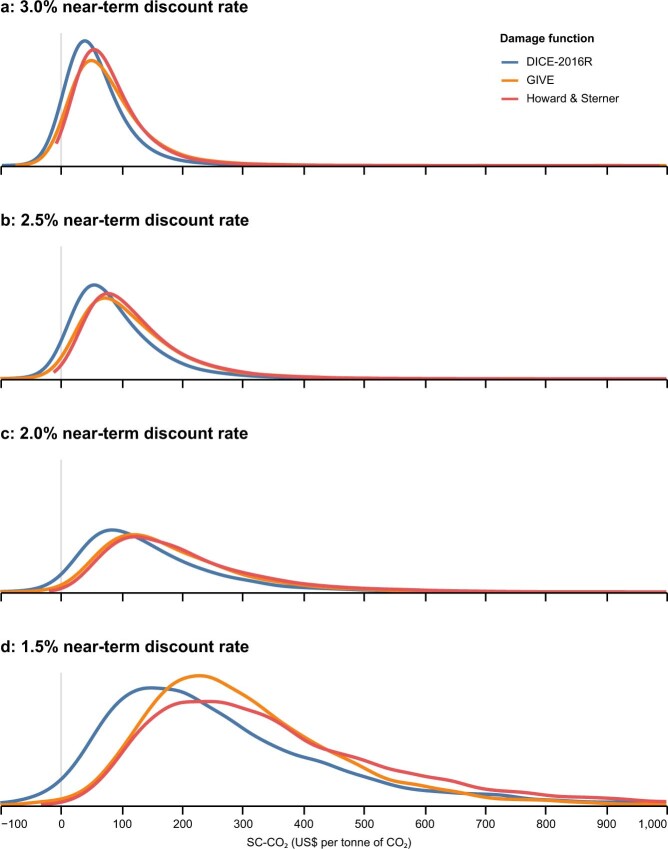
SC-CO_2_ distributions are robust to different damage function specifications ($ per tCO_2_). Distributions of the SC-CO_2_ using the damage functions from GIVE (orange, our preferred specification), DICE-2016R^32^ (blue), and Howard & Sterner^33^ (red) for near-term discount rates of 1.5%, 2.0%, 2.5% and 3.0%. All results use the RFF-SP scenarios, a stochastic growth-linked discounting framework, and sample uncertain climate, sea-level and damage function parameters, including for DICE-2016R and Howard & Sterner^33^ damage functions. The DICE-2016R damage function is based on ref. ^32^ (see page 2 of that work’s supporting information)^32^. The Howard & Sterner damage function is based on the base coefficient in their table 2, specification (8). All SC-CO_2_ values are expressed in 2020 US dollars per metric tonne of CO_2_.

**Extended Data Fig. 2:**
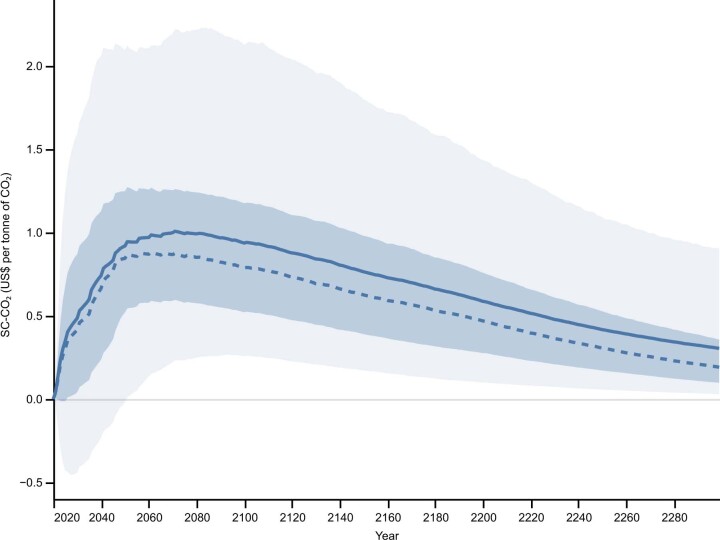
Discounted marginal damages by year, preferred 2% near-term discount rate case. Solid line represents mean discounted marginal damages for a one-tonne CO_2_ emissions pulse in 2020, dotted line represents the median, with darker shading spanning the 25%–75% quantile range and lighter shading spanning the 5%–95% quantile range. All SC-CO_2_ values are expressed in 2020 US dollars per metric tonne of CO_2_.

We also assess (Extended Data Fig. 1 and Table 1) the sensitivity of our SC-CO_2_ estimates to discounting by also using near-term rates of 3% ($80 per tCO_2_ mean, $12–$197 per tCO_2_: 5%–95% range), to facilitate comparison with the US government’s current, most commonly cited $51 per tCO_2_ figure, as well as 2.5% ($118 per tCO_2_ mean, $23–$280 per tCO_2_: 5%–95% range) and 1.5% ($308 per tCO_2_ mean, $94–$626 per tCO_2_: 5%–95% range). We additionally show (Extended Data Fig. 2) the temporal evolution of the discounted marginal damages by year based upon the preferred 2% near-term discount rate case.

**Table 1:** Evolution of mean SC-CO_2_ from DICE-2016R to this study

Row	Scenario	Mean SC-CO_2_ ($ per tCO2)	Incremental change ($ per tCO2)	Share of total change (%)
a	DICE-2016R	44		
b	GIVE with DICE damage function, 3% near-term discount rate	59	15	11
c	GIVE with sectoral damages, 3% near-term discount rate	80	21	15
d	This study: GIVE with sectoral damages, 2% near-term discount rate	185	105	74

All SC-CO_2_ values are expressed in 2020 US dollars per metric tonne of CO_2_. Row a represents the SC-CO_2_ using base DICE-2016R deterministic. The mean SC-CO_2_ of $44 per tCO_2_ is similar to the value previously estimated from IWG DICE-2010 of $46 per tCO_2_ at a 3% discount rate, after converting to 2020 dollars^65^. Row b then retains the DICE-2016R damage function but otherwise deploys GIVE under discounting parameters of *ρ* = 0.8%, *η* = 1.57, which are consistent with a 3% near-term discount rate (see Methods section ‘Discounting’ for descriptions of *ρ* and *η*). Row c replaces the DICE-2016R damage function with our sectoral damage functions, and row d then uses our preferred discounting parameters from this study of *ρ* = 0.2%, *η* = 1.24, which are consistent with a 2% near-term discount rate. The final row represents the preferred mean value from this study.

Our SC-CO_2_ estimates are based on regionally disaggregated damage functions for four sectors. As a sensitivity analysis, we replace the sectoral damage functions in GIVE with two distinct, globally aggregated damage functions that are based on meta-analyses of the climate impact literature^32,33^. Under a 2% near-term discount rate, these sensitivity runs yield relatively similar SC-CO_2_ distributions with mean values that differ by −18% to +11% (Extended Data Table 1) from our preferred SC-CO_2_ estimate (Extended Data Fig. 1).

The single largest contributor to the overall increase in the SC-CO_2_ relative to the widely used DICE model is the use of a lower near-term discount rate, and updated damage functions are the second largest contributor. We disaggregate impacts of the changes to the near-term discount rate, the sectoral damage functions, and the remaining GIVE components (the RFF-SPs and FaIR) in Table 1. We start by running DICE-2016R, which uses none of our updated components and uses DICE’s default discounting approach, yielding an SC-CO_2_ estimate of $44 per tCO_2_. Updating the climate modelling, the socioeconomic scenarios, and the discounting approach reflecting a 3% near-term discount rate but retaining the DICE-2016R damage function increases the mean SC-CO_2_ by 34% to $59 per tCO_2_. Incorporating our sectoral damage functions in place of the DICE-2016R damage function further increases the estimate to $80 per tCO_2_, or a total increase of 81%. Finally, using a lower 2% near-term discount rate has the largest effect, increasing the mean SC-CO_2_ estimate to this study’s value of $185 per tCO_2_, a 321% increase relative to $44 per tCO_2_, and a 3.6-fold increase relative to the widely cited US government value of $51 per tCO_2_.

**Fig. 3:**
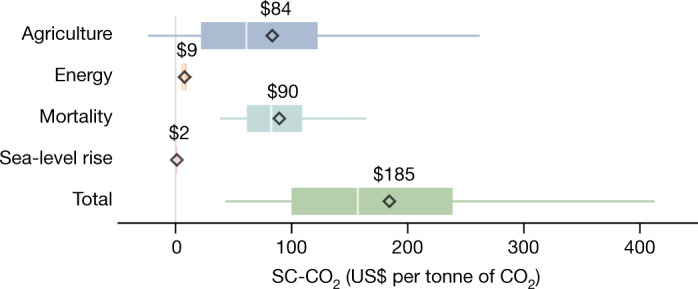
Partial SC-CO_2_ estimates and uncertainty levels strongly differ across the four climate damage sectors. Box and whisker plots for the climate damage sectors included in the GIVE model, based on partial SC-CO_2_ estimates for each sector. The figure depicts the median (centre white line), 25%–75% quantile range (box width), and 5%–95% quantile range (coloured horizontal lines) partial SC-CO_2_ values. Black diamonds highlight each sector’s mean partial SC-CO_2_, with the numeric value written directly above. All SC-CO_2_ values are expressed in 2020 US dollars per metric tonne of CO_2_.

The four climate damage sectors represented in the model vary substantially in their respective contributions to the overall magnitude and uncertainty of the SC-CO_2_ (Fig. 3). Temperature mortality impacts are the largest driver of the SC-CO_2_, contributing a mean partial SC-CO_2_ (defined as the SC-CO_2_ estimated for an individual impact sector) of $90 per tCO_2_ ($39–$165 per tCO_2_: 5%–95% range) to the $185 per tCO_2_ total using a near-term 2% discount rate. Agricultural impacts have a similar mean contribution of $84 per tCO_2_, but greater uncertainty, with a 5%–95% partial SC-CO_2_ range spanning −$23 to $263 per tCO_2_. This large range, which includes the potential for beneficial effects of higher temperatures and CO_2_ concentrations on agriculture, arises owing to compounding uncertainty in the relationship between CO_2_, temperature and crop yields, and how these factors interact with the economic system to affect human welfare^18^. We sample uncertain parameters for mortality and agriculture (see Methods), the damage sectors for which parameter uncertainty is quantified in the underlying studies.

The relatively small contribution of sea-level rise, which includes both coastal damages and adaptation costs, to the total SC-CO_2_ (mean partial SC-CO_2_ of $2 per tCO_2,_ $0–$4 per tCO_2_: 5%–95% range) is attributable in part to the inertia in the physical system connecting CO_2_ emissions and sea-level rise and in part to the optimal regional adaptation response allowed by the Coastal Impact and Adaptation Model (CIAM) that we incorporate into GIVE^21^. Such optimal, forward-looking adaptation responses can substantially reduce estimated coastal damages relative to a static scenario assuming no response to evolving coastal risks^55,56^. Future research could improve the characterization of plausible versus optimal coastal adaptation responses. The relatively slow pace of sea-level rise also causes the greatest damages to occur far in the future when discounting effects are strongest. Energy costs for residential and commercial buildings (based on a previous work)^20^ also make a relatively small contribution to the overall SC-CO_2_ (mean partial SC-CO_2_ of $9 per tCO_2,_ $4–$15 per tCO_2_: 5%–95% range), owing to increased energy demand from cooling being offset by decreased heating demand and future technological progress; these results are broadly consistent with other recent empirical work^57^.

We quantify the impact on four critical, globally significant damage sectors that are often considered to contribute the most to the SC-CO_2_^1,58^ and for which studies exist that can be readily incorporated into SC-CO_2_ estimation owing to their global coverage, regional disaggregation and monetization. A limitation of this study is that other categories of climate damages—including additional non-market damages other than human mortality—remain unaccounted for. The inclusion of additional damage sectors such as biodiversity^59^, labour productivity^60,61^, conflict^62^ and migration^63^ in future work would further improve our estimates. Current evidence strongly suggests that including these sectors would raise the estimates of the SC-CO_2_, although accounting for adaptation responses could potentially counteract some of that effect. Other costs of climate change, including the loss of cultural heritage, particular ways of life, or valued ecosystems, may never be fully valued in economic terms but would also probably raise the SC-CO_2_ beyond the estimates presented here. The addition of alternate studies covering the same sectors to incorporate additional independent lines of evidence is also a promising area for continued work to improve the SC-CO_2_. The modular structure of the Mimi.jl framework facilitates such addition of new damage sectors with ease, providing a flexible basis for future scientific improvement of the SC-CO_2_.

Although we approximate the effects of a rapid Antarctic ice sheet disintegration tipping point within the BRICK sea-level component, incorporating additional potential discontinuities in the climate system would further improve our SC-CO_2_ estimates^64^. We expect that, in total, the future inclusion of additional damage sectors and tipping elements will probably raise the estimates of the SC-CO_2_, and therefore that the estimates from the present study are probably best viewed as conservative. Similarly, accounting for different climate model structures, as the recent IPCC AR6 report does in chapter 7^14^, would further strengthen the robustness of our SC-CO_2_ estimates and their associated uncertainty levels. For example, that chapter (see cross-chapter box 7.1 and table 2)^14^ shows that the MAGICC climate model projects slightly higher temperature increases than the FaIR model.

The methods used in this study reflect the culmination of several important advances: development of fully probabilistic very-long-run socioeconomic inputs that natively incorporate uncertainty over future climate policy; incorporation of state-of-the-science representations of the climate system and sectoral damage functions; and an empirically calibrated discounting approach that accounts for uncertainty in future economic growth. These advances collectively allow for the full characterization of uncertainties, and their compounding interactions, throughout all steps of SC-CO_2_ estimation, including sectoral market and nonmarket damages to human health. Their implementation on Mimi.jl^8^, an open-source, modular computational platform for assembling IAMs, improves the scientific basis and transparency of the resulting estimates and is responsive to the NASEM near-term recommendations. The methodology also provides a straightforward means with which to calculate SC-CO_2_ results for other years and estimate the social cost of other greenhouse gases (for example, CH_4_, N_2_O and hydrofluorocarbons). Our higher SC-CO_2_ values, compared to estimates currently used in policy evaluation, substantially increase the estimated benefits of greenhouse gas mitigation, and thereby increase the expected net benefits of more stringent climate change policies.

## Methods

### Socioeconomic projections

The RFF-SPs^3^ used in this study were designed to address the requirements for socioeconomic projections posed by SC-CO_2_ estimation: (1) The roughly 300-year time horizon required to account for the vast majority of discounted future damages; (2) the need for geographically disaggregated estimates of GDP and population to support damages at a regional scale; (3) uncertainty accounting for expected future changes in both technology and policy (the SC-CO_2_ is measured against the best estimate of future emissions, inclusive of future mitigation policies except the one under analysis); and (4) the interdependence of future population, GDP and greenhouse gas emissions trajectories^1^.

The RFF-SPs address key shortcomings identified in the approach to socioeconomic projections originally developed by the US IWG in 2010^66^ and used consistently through the current US interim estimates^43^. The IWG used five socioeconomic scenarios to 2100, drawn from the Energy Modeling Forum 22 modelling exercise^67^, one of which represented future climate policy. The IWG scenarios were critiqued for not spanning the true uncertainty in GDP, population and emissions, nor reflecting the broader scenario literature overall^34,68^. The RFF-SPs used here improve on those scenarios by explicitly characterizing uncertainty in the demographic, economic and emissions projections.

The multi-century time horizon required for the projections is long relative to the length of the historical record available to estimate country-level statistical models of population and economic growth. Accounting for uncertainty in future emissions over that time horizon requires assessing the potential for structural changes in technology and policies that are out of the range of historical experience. To address these challenges, the RFF-SPs were generated based upon a combination of statistical and expert-based approaches.

We generated probabilistic, country-level population projections through 2300^9^ by extending the fully probabilistic statistical approach used by the United Nations for its official population forecasts to 2100. We further incorporated feedback and improvements suggested by a panel of nine leading demographic experts convened to review preliminary results.

Our trajectories of country-level GDP per capita from 2018 to 2300 come from a multifactor Bayesian dynamic model, in which each country’s GDP per capita is based on a global frontier of developed economies and country-specific deviations from that frontier^10^. We reweight the probabilities of the Bayesian model trajectories using results from the RFF Economic Growth Survey, a formal expert elicitation focused on quantifying uncertainty in long-run economic growth^3^.

The resulting probabilistic socioeconomic trajectories represent an alternative to existing scenario-based approaches, such as those based on the Shared Socioeconomic Pathways narratives. Such scenarios do not typically come with associated probabilities, though there have been efforts to assign such probabilities to the SSPs a posteriori on the basis of expert surveys^37^. The use of non-probabilistic scenarios have been criticized in the literature for being overconfident and failing to reflect uncertainty^69^. Indeed, multi-century socioeconomic projections are deeply uncertain, as illustrated by the wide 5%–95% ranges that we consider (see Fig. 1). The scenarios based on the SSP narratives and their commonly used extensions beyond 2100^63,70–72^ fail to span that uncertainty^3^.

We also generate multi-century distributions of global CO_2_, CH_4_ and N_2_O emissions through RFF’s Future Emissions Survey, which elicited experts in socioeconomic projections and climate policy^3^. Experts provided uncertainty ranges for future fossil fuel and process-related CO_2_ emissions as well as changes in natural CO_2_ stocks and negative-emissions technologies, incorporating their own uncertainty around future mitigation policy. They also quantified the sensitivity of emissions projections to future economic growth, thereby allowing for the development of a joint set of projections of emissions and economic growth. The experts additionally provided uncertainty ranges for trajectories of CH_4_ emissions, N_2_O emissions, and net CO_2_ emissions from other sources of CO_2_ emissions and sinks.

### Climate models

#### FAIR

We represent the global climate system and carbon cycle dynamics using version 1.6.2 of the Finite Amplitude Impulse Response (FaIR) model^73–75^. FaIR is an emissions-based simple climate model with a carbon cycle that depends on background warming levels and cumulative carbon uptake by land and ocean sinks. This state-dependency enables FaIR to replicate the equilibrium and impulse-response behaviours found in more sophisticated Earth system models, which is important for producing scientifically grounded SC-CO_2_ estimates. These features are not found in the previous climate models used for SC-CO_2_ calculations, which lack carbon cycle feedback and have been shown to respond too slowly to changes in radiative forcing^1,11^. We run FaIR with randomly sampled CO_2_, CH_4_ and N_2_O emissions time series from the RFF-SPs and represent other greenhouse gases and short-lived climate forcers using the SSP2-4.5 scenario^76^, which is the scenario that most closely matches the median RFF-SP emissions trajectories. We account for climate model uncertainties by randomly sampling a calibrated 2,237-member ensemble of parameters that was produced using FaIR as part of the IPCC AR6^74^. See Supplementary Information section SI.2 for more detail on the FaIR model.

#### BRICK

We make probabilistic projections of regional changes in sea level using the Building blocks for Relevant Ice and Climate Knowledge (BRICK) model. BRICK represents individual contributions to sea level from the Greenland and Antarctic ice sheets, glaciers and small ice caps, thermal expansion, and land water storage and has been thoroughly described in prior studies^15^. BRICK downscales changes in global sea level to regional changes using maps of time-invariant scaling factors^15,77^. The Antarctic ice sheet model component also accounts for a potential tipping point where rapid ice sheet disintegration can occur when annual mean Antarctic surface temperatures cross an uncertain threshold^16^.

We closely follow past work and calibrate BRICK to the historic sea-level record over the period 1850–2017 with a Bayesian framework^15,17,78,79^. This calibration process uses observational constraints on global mean sea-level changes^80^ in addition to individual contributions from glaciers and small ice caps^81^, the Greenland ice sheet^82,83^, the Antarctic ice sheet^84^ and trends in thermal expansion^85^. It further statistically accounts for measurement error estimates provided with each observational time-series dataset^86^. We select physically informed prior distributions for BRICK’s uncertain parameters that are consistent with previous model calibration studies^15,17^. For the Antarctic ice sheet model component, we select prior distributions based on a paeleoclimate calibration that uses independent sea-level data from 240,000 years before the current era to the present^16^. We use our calibration framework to create a Markov chain of ten million representative samples from BRICK’s joint posterior parameter distribution and assess convergence based on graphical diagnostics and Gelman–Rubin potential scale reduction factors that are less than 1.1^87,88^. We discard the first one million samples for the initial burn-in period and select a random subset of 10,000 samples from the remaining chain for our final sea-level parameter values. The distributions of the uncertain parameters in BRICK are shown in Supplementary Information Table 4.

### Damage functions

#### Sea-level rise

The sea-level rise damage calculations are based on a previous work^21^ that presents the Coastal Impacts and Adaptation Model (CIAM). CIAM is an optimization model that assesses the costs of various adaptation strategies against flooding damages and potential impacts from regional changes in sea level. It chooses the least-cost strategy for each of over 12,000 coastal segments across the globe in the Dynamic Interactive Vulnerability Assessment (DIVA) database^89^ after taking into account local physical and socioeconomic characteristics. CIAM’s potential adaptation strategies are specified as a combination of (1) a choice on retreating inland from the coastline, protecting coastal communities and infrastructure, or remaining in place without taking any adaptive actions and (2) a choice on the degree of investment in coastal defence against several different storm surge return periods conditional on protection being decided on. The DIVA database provides generalized extreme value distributions that define these return periods for each individual segment.

CIAM is a deterministic model. All uncertainty in coastal damages is therefore the result of uncertain sea-level projections that arise owing to GIVE’s probabilistic emission scenarios and climate and sea-level model parametric uncertainties that we sample.

#### Building energy expenditures

The energy demand damage function is based on the results of Clarke et al. 2018^20^, a study that used the Global Change Analysis Model (GCAM)^90,91^ to project how climate change affects regional building energy demand through 2100. GIVE’s damage functions relate each degree of global temperature rise to a change in regional energy expenditures, expressed as a proportion of that region’s GDP. We derive these damage functions using output data provided by the authors of ref. ^20^. That output includes, for each of the 12 GCAM regions, the net change in regional energy expenditures as a proportion of regional GDP at various temperature levels (varying over both time and scenario). Reference ^20^ notes that this relationship is approximately linear in temperature. For each of the 12 GCAM regions, we fit a linear function to these datapoints by regressing the net change in energy expenditures as a proportion of GDP on global temperature rise relative to the preindustrial period. We assume the intercept is zero to ensure the resulting function yields no change in energy expenditures at zero temperature rise. This yields a coefficient for each region, denoted ${\beta }_{j}^{E}$ (see Supplementary Information Table 2 for these values). Energy damages for each country *i* located in region *j* are then calculated using the corresponding coefficient, as

1 $${\text{Change in energy expenditures as a proportion of GDP}}_{i,t}={\beta }_{j}^{E}\times {(\text{Temperature rise})}_{t}.$$

We multiply this energy expenditure share by country-level GDP to generate damages in dollars.

Reference ^20^ did not feature any explicit consideration of uncertainty, so we do not include uncertainty in this damage function. Uncertainty in energy-related damages remain, however, owing to GIVE’s uncertain temperature projections and GDP trajectories.

#### Temperature-related mortality

The mortality damage functions are based on the results of Cromar et al. 2022^19^, in which a panel of health experts was convened to conduct a meta-analysis of peer-reviewed research studying the impacts of temperature on all-cause mortality risk, which includes human health risks related to a broad set of health outcomes including cardiovascular, respiratory and infectious disease categories. The meta-analysis combined studies to produce regionally disaggregated estimates of the effects on all-cause mortality of each degree of warming across a broad range of baseline temperatures, including both increased mortality risk at high temperatures and reduced risk at cooler temperatures. This produced, for each of 10 regions, a point estimate (and its standard error) representing the net change in all-cause mortality risk per degree Celsius of globally averaged surface temperatures (see Supplementary Information Table 1).

To reflect uncertainty in these estimates, we sample these parameters ${\beta }_{j}^{M}$ for region *j* from a normal distribution centred on the point estimate and set the standard deviation equal to the reported standard error. We then compute temperature-induced excess deaths in country *i* in region *j* as

2 $${(\text{Temperature-induced excess deaths})}_{i,t}={\beta }_{j}^{M}\times {(\text{Temperature rise})}_{t}\times {(\text{Baseline mortality})}_{i,t},$$

where we calculate baseline mortality as the regional population level times its baseline mortality rate from the RFF-SPs,

3 $${(\text{Baseline mortality})}_{i,t}={\text{Population}}_{i,t}\times {(\text{Baseline mortality rate})}_{i,t}.$$

We monetize these excess deaths using the value of a statistical life (VSL) as follows:

4 $${({\rm{Monetized\; excess\; mortality}})}_{i,t}={{\rm{VSL}}}_{i,t}\times {({\rm{Temperature-induced\; excess\; deaths}})}_{i,t}.$$

The baseline VSL value for 2020 for the USA (denoted $${{\rm{VSL}}}_{{\rm{US}},2020}^{{\rm{base}}}$$) is derived using EPA’s 1990 Guidance value of $4.8 million and adjusted for income growth and inflation, resulting in a 2020 US VSL of $10.05 million in 2020 dollars^44^ (see data explainer notebook in the replication code for this paper for the full derivation). We then base the VSL for country *i* in year *t* on the EPA’s baseline VSL for 2020, adjusted for country *i*’s GDP per capita in year *t*, as

5 $${{\rm{VSL}}}_{i,t}={{\rm{VSL}}}_{{\rm{US}},2020}^{{\rm{base}}}\times {\left(\frac{{{\rm{GDP\; per\; capita}}}_{i,t}}{{{\rm{GDP\; per\; capita}}}_{{\rm{US}},2020}}\right)}^{\varepsilon },$$

where *ε* = 1 represents the income elasticity of the VSL. The primary function of *ε* is to adjust the US VSL to other countries and at uncertain future income levels. We use a unit elasticity, which is in line with the central tendency of values recommended in the literature for such cases^92–95^.

#### Agriculture

The agricultural damage function is based on Moore et al. 2017^18^, which estimated damages in two steps using: (1) a meta-analysis of published studies of the effects of temperature, rainfall and CO_2_ on crop yields that builds on previous work^96,97^, and (2) a computable general equilibrium model to estimate the economic welfare consequences of these yield shocks while accounting for trade patterns and supply and demand adjustments in agricultural markets across 16 regions.

Reference ^18^ presents results in the form of damage functions that directly relate global mean surface temperature increase to welfare change in economic terms. Their study presents three different parameterizations of these damage functions to characterize uncertainty: a central, low and high estimate.

They estimated each of these three parameterizations for 1, 2 and 3 degrees Celsius of temperature increase, resulting in three piecewise linear damage functions for each region (see Supplementary Information Fig. 1). To address uncertainty as part of our Monte Carlo sampling framework, we sampled a value from a triangular distribution with lower bound 0, mode 0.5 and upper bound 1 for each draw. Assigning the low, central and high damage functions to each of these values respectively, the two nearest functions were linearly interpolated to produce the damage function for that draw, also interpolating linearly between the resultant 1-degree Celsius value and the origin, since damages at zero temperature increase can be assumed to be zero. Importantly, this uncertainty sampling scheme preserves the covariance between regions arising through connections in the global trade network.

Lastly, we incorporated their results into our model via the equation

$${{\rm{AgPctCost}}}_{i,t}=\mathop{\underbrace{{\sigma }_{i}{\left(\frac{{{\rm{G}}{\rm{D}}{\rm{P}}{\rm{p}}{\rm{e}}{\rm{r}}{\rm{c}}{\rm{a}}{\rm{p}}{\rm{i}}{\rm{t}}{\rm{a}}}_{i,t}}{{{\rm{G}}{\rm{D}}{\rm{P}}{\rm{p}}{\rm{e}}{\rm{r}}{\rm{c}}{\rm{a}}{\rm{p}}{\rm{i}}{\rm{t}}{\rm{a}}}_{i,1990}}\right)}^{-\epsilon }}}\limits_{{\rm{a}}{\rm{g}}\,{\rm{s}}{\rm{h}}{\rm{a}}{\rm{r}}{\rm{e}}}{f}_{i}({T}_{t}),$$

where AgPctCost_*i*,*t*_ is the damage in the agricultural sector as a proportion of GDP in region *i* at time *t*; *σ*_*i*_ is the share of agriculture in GDP in 1990 in region *i*; *ϵ* = 0.31 is the income elasticity of the agriculture share in GDP^98^; *T*_*t*_ is global average surface temperature increase; and *f*_*i*_ is the piecewise linear function for region *i* resulting from the steps described above.

### Discounting

Our discounting approach directly follows from NASEM recommendations as developed in a previous work^1,31^. Given the long residence time of CO_2_ in the atmosphere, the damages from CO_2_ emitted today persist for centuries. These future damages must be converted to present dollar equivalents using an appropriate discount rate. The climate economics literature typically uses Ramsey-style discounting that links the discount rate to future economic growth^99^. This linkage leads to the Ramsey-like equation for the discount rate over time, denoted *r*_*t*_: *r*_*t*_ = *ρ* + *ηg*_*t*_, where *ρ* is the rate of pure time preference, *g*_*t*_ is the average rate of consumption growth from the year of the emissions pulse (described in the next section) to year *t*, and *ηg*_*t*_ reflects the extent to which society discounts damages because future individuals are relatively wealthier. More specifically, *η* reflects how much the marginal value of consumption declines as consumption increases (a 1% increase in consumption corresponds with a *η*% decline in the marginal value of a dollar).

We evaluate the stochastic discount rate for each realized path of uncertain consumption growth (*r*_*t*_ = *ρ* + *ηg*_*t*_), explicitly and structurally modelling the uncertainty in discount rates that is often summarized by a declining term structure^100^. This uncertainty in the discount rate leads to a stochastic discount factor (SDF_*t*_) used to discount future marginal climate damages. The SDF_*t*_ can also be written equivalently in terms of relative consumption levels^54,101^ as

6 $${{\rm{S}}{\rm{D}}{\rm{F}}}_{t}=\frac{1}{{(1+\rho )}^{t-2020}}{\left(\frac{{c}_{t}}{{c}_{2020}}\right)}^{-\eta }.$$

Here *c*_*t*_ is world average per capita consumption in year *t*. We use this SDF_*t*_ to discount marginal climate damages (MD_*t*_) to a present value.

Whereas the climate economics literature routinely uses a Ramsey-like approach to discounting^32,54,101–105^, prior estimates by the US IWG disconnected discounting and future economic growth by using a constant, deterministic discount rate. That approach implicitly assumes that *η* = 0, corresponding to no linkage between consumption growth and discounting as well as zero aversion to risk. Our approach re-establishes the Ramsey-like link between growth and discount rates. We use *ρ* and *η* values that were empirically calibrated^3^ to be consistent with the RFF-SPs and evidence on the observed behaviour of interest rates^48^. This procedure also produces near-term risk-free discount rates (defined as the average risk-free discount rate over the first decade of the time horizon) consistent with the desired values, such as those reported in Fig. 1. Our preferred SC-CO_2_ estimate corresponds to a near-term 2% rate, which is consistent with real risk-free interest rates over the last 30 years, and uses *ρ* = 0.2% and *η* = 1.24 (refs. ^3,31^). The (*ρ*, *η*) values corresponding to the alternative near-term rates of 1.5%, 2.5% and 3% are (0.01%, 1.02), (0.5%, 1.42) and (0.8%, 1.57), respectively.

The Ramsey-like form for the discount rate is a standard approach to value marginal impacts and account for their risk amid uncertainty in future payoffs and consumption levels in the discounted expected utility framework^53,54^. In that framework, the value of the *η* parameter reflects the degree of risk aversion as well as the inverse of the intertemporal elasticity of substitution. That framework is also used for benefit–cost analysis of policy and regulatory analysis under uncertainty, as it quantifies the risk premium associated with uncertainty and risk aversion in the valuation of a marginal emission of CO_2_. Although the Ramsey framework is widely used, other considerations for decision-making under uncertainty in the context of climate change, such as the role of epistemic uncertainty and alternative preference structures including ambiguity aversion, have also been proposed^106^. We use the discounted expected utility framework because it is the most established and widely used framework for regulatory and policy analysis^107,108^.

### Estimating the SC-CO_2_

We estimate the SC-CO_2_ in a three-step calculation process. In the first step, we run the GIVE model out to the year 2300 for two separate cases: a ‘baseline’ case and a ‘perturbed’ case that adds an extra 0.1 MtC pulse of CO_2_ emissions in the year 2020 and is otherwise identical. In the second step, we calculate marginal climate damages in year *t* as the difference in modelled damages per tonne between the pulse and baseline runs as

7 $${{\rm{M}}{\rm{D}}}_{t}=\mathop{\sum }\limits_{d=1}^{4}\mathop{\sum }\limits_{r=1}^{{R}_{d}}({\text{Damages with pulse}}_{t,d,r}-{\text{Baseline damages}}_{t,d,r}),$$

where we aggregate over each of the four damage sectors *d* at their respective geographic resolutions (that is, countries or regions) *r*.

In the third and final step, we calculate the SC-CO_2_ by discounting these marginal damages using the stochastic discount factors SDF_*t*_ from equation (5) above and then aggregate them over time into a single present value

8 $${{\rm{SC-CO}}}_{2}=\mathop{\sum }\limits_{t=2020}^{2300}{{\rm{SDF}}}_{t}\times {{\rm{MD}}}_{t}.$$

For our preferred results, we calculate 10,000 unique SC-CO_2_ estimates. For each estimate, we sample the RFF-SP scenarios to account for uncertainties in global CO_2_, CH_4_ and N_2_O emission trajectories in addition to country-level population and GDP growth levels. We also sample parametric uncertainties in the FaIR and BRICK models as well as the agricultural and temperature-related mortality damage functions (Extended Data Table 2). As described above, our preferred SC-CO_2_ estimate uses discounting parameters of *ρ* = 0.2% and *η* = 1.24 for a near-term rate of 2%.

When we report partial SC-CO_2_ estimates for a given damage sector, we follow the estimation procedure outlined above, but only include the impacts from that individual sector when calculating marginal damages in equations (7), (8). We normalize our estimates on the basis of emission pulse size and report all results throughout the paper in units of 2020 US dollars per metric tonne of CO_2_. We use the implicit GDP price deflator from the US Bureau of Economic Analysis to convert values to 2020 dollars.

We typically summarize the distribution of our 10,000 SC-CO_2_ estimates by its mean, that is, *E*[SC-CO_2_], where the expectation operator is taken jointly over all uncertain parameters determining marginal damages (MD_*t*_) and the stochastic discount factor (SDF_*t*_). This calculation is consistent with economic theory for pricing investments and other actions with uncertain payoffs, and therefore properly accounts for the risk premium in the valuation of a marginal emission of CO_2_ owing to the many compounding uncertainties we model^46^.

### Software

All our results are computed using open-source software tools. We use the Julia programming language for the entire replication code of this paper^109^. All models used in this study are implemented on the Mimi.jl computational platform for integrated assessment models^8^.

## Online content

Any methods, additional references, Nature Research reporting summaries, source data, extended data, supplementary information, acknowledgements, peer review information; details of author contributions and competing interests; and statements of data and code availability are available at 10.1038/s41586-022-05224-9.

### Supplementary information

Supplementary Information This file contains Supplementary Methods and Discussion; Supplementary Tables; Supplementary Figures and Supplementary References.

## Figures and Tables

**Extended Data Table 1:**
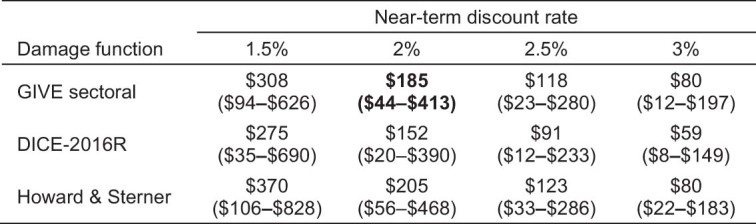
Mean SC-CO_2_ values (with 5th–95th quantile ranges), by damage function and discount rate ($ per tCO_2_)

Our preferred estimates correspond to the GIVE sectoral damage functions at a 2% near-term discount rate, shown in bold. All results use the RFF-SP scenarios, a stochastic growth-linked discounting framework, and sample uncertain climate, sea level, and damage function parameters, including for DICE-2016R and Howard & Sterner^33^ damage functions. The DICE-2016R damage function is based on Nordhaus 2016 (see page 2 of that work’s supporting information)^32^. The Howard & Sterner damage function is based on the base coefficient in their table 2, specification (8). All SC-CO_2_ values are expressed in 2020 US dollars per metric tonne of CO_2_.

**Extended Data Table 2:**
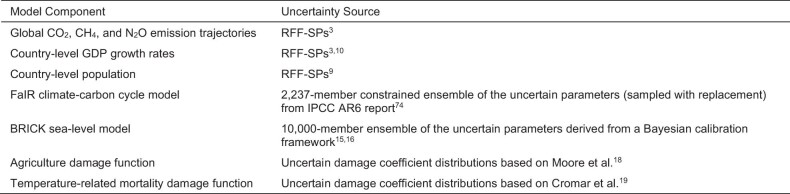
Sources of SC-CO_2_ uncertainty

The left column shows the inputs and components of the GIVE model that contribute to uncertainty in the SC-CO_2_. The right column briefly describes these uncertainties and their sources. Refs. ^3,9,10,15,16,18,19,74^.

## Data Availability

Data for this paper are available at 10.5281/zenodo.6932028.

## Code availability

The replication code for this paper is available at 10.5281/zenodo.6932028, including instructions on how to rerun the entire analysis for this paper.
