# Supplementary material for: Comprehensive evidence implies a higher social cost of CO2
Source: Nature. 2022 Sep 1;610(7933):687–92. doi: 10.1038/s41586-022-05224-9 (PMC9605864; doi:10.1038/s41586-022-05224-9)
Supplement: Supplementary file 1 — This file contains Supplementary Methods and Discussion; Supplementary Tables; Supplementary Figures and Supplementary References. [file 41586_2022_5224_MOESM1_ESM.pdf]

nature portfolio

https://doi.org/10.1038/s41586-022-05224-9

Supplementary information

Comprehensive evidence implies a higher social cost of CO_2

In the format provided by the authors and unedited

Supplementary Information for "Comprehensive Evidence Implies a Higher Social Cost of CO_2" by Rennert et al.

SI.1 Methods and Discussion: RFF Socioeconomic Projections (RFF-SPs)

The RFF-SPs are an internally consistent set of probabilistic projections of country-level population[9] and per-capita economic growth,[3,10] and global CO_2, CH_4, and N_2O emissions[3] generated using a combination of statistical modelling and expert elicitation. The form of expert elicitation used to generate the RFF-SPs, referred to as "Structured Expert Judgement", has been described in previous research[110–112]. Our application of Structured Expert Judgment to the SC-CO_2 is novel in several respects: (i) for the first time it employs traceable and validated quantitative expert uncertainty quantification, (ii) constructing a complex multivariate stochastic process out to 2300 encompassing CO_2 emissions, GDP growth, and population which (iii) leverages near-term data driven population and GDP models (iv) capturing regional differences while (v) for the first time accounting for dependence between the variables. The full methodology used to create the RFF-SPs individual components, briefly described below, is detailed in full in Rennert et al. (2021)[3] The RFF-SPs are freely available at https://doi.org/10.5281/zenodo.5898729.

Population:

The RFF-SPs employ the country-level population projections to the year 2300 detailed in Raftery and Ševčíková.[9] These projections were generated by extending the fully probabilistic statistical method used by the UN for its official forecasts to 2100. This method combines Bayesian hierarchical models for each of the three components of population change: fertility, mortality, and international net migration, each estimated using Markov chain Monte Carlo (MCMC) methods. These generate many trajectories of future values of the population change components for each country, and each one then yields a trajectory of future population for all countries and periods. The modifications made to extend the model to 2300 incorporated specific improvements suggested by a panel of nine leading demographic experts convened to review preliminary estimates. In accordance with the panellists' suggestions, the extended approach

incorporates a worldwide random walk component to the total fertility rate model, providing a lower bound of 1.2 children per woman for the world in 2300. It also implements international migration probabilistically using a Bayesian hierarchical model[113,114] which represents an improvement on the deterministic approach taken for the UN projections. Constraints are imposed on population density to prevent unrealistically high or low population numbers in some age groups in some countries.

Economic growth

To generate country level projections of economic growth per capita to the year 2300, the RFF-SPs utilize a statistical approach for projecting country-level economic growth augmented with the results from a structured expert judgement study tailored for this purpose.[3]

The statistical approach, described in Müller, Stock, and Watson (2020)[10], henceforth MSW, utilizes a multifactor Bayesian dynamic model in which each country's GDP per capita is based on a global frontier of developed economies (countries in the Organisation for Economic Co-operation and Development, OECD) and expressed as country-specific deviations from that frontier. The MSW model captures correlations between countries via a hierarchical structure that allows county-level deviations from the frontier to vary together via "covariance clubs" and for the pooling of information across countries. It is estimated on data for 113 countries over 118 years (1900 to 2017) to yield 2,000 sets of trajectories of country-level GDP per capita from 2018 to 2300.

Each set of trajectories is characterized by a path for the global factor and 113 country-specific deviations from that pathway and can be considered an equally likely uncertain future.

In order to quantify uncertainty in economic growth over the very long time-horizon, Resources for the Future conducted its Economic Growth Survey (EGS) to augment the historical statistical evidence from MSW. The EGS was a structured expert judgment study that quantified uncertainty in future economic growth per capita for OECD countries. The EGS was designed generally to be used with projections based on an evolving frontier of growth and was specifically applied to the results of the MSW model. The resulting dataset provides country-level, long-run probabilistic projections of economic growth per capita informed by both the statistical and expert approaches.

The "classical model"[115,116] was applied for validating and combining expert judgments. Experts are treated as statistical hypotheses and scored with respect to statistical accuracy and informativeness based on responses to calibration variables from their field to which the true values are available post hoc. Performance-based weights are used to combine the experts' distributions in a linear pool. The weights are asymptotically strictly proper scoring rules, that is, in the long run, an expert achieves their maximal expected weight by and only by stating their true opinion. The resulting combination is also scored on the calibration variables and compared with an equal weight combination. The performance based combination has been shown superior both in- and out-of-sample and results in distributions with narrower overall uncertainty distributions and good statistical accuracy [117–119]

The EGS elicited ten experts that had been selected for their expertise in macroeconomics and economic growth and recommended by their peers, individually by videoconference in roughly two-hour interviews in 2019–2020. The experts first quantified their uncertainty for several initial questions, after which answers were provided for self-assessment to familiarize them with the process and to alert them to potential biases. The experts then provided a median and 90 percent confidence range for 11 calibration questions for which the true values were known to those administering the survey. Experts next provided their 1st, 5th, 50th, 95th, and 99th quantiles for the levels of OECD GDP per capita for 2020-2050, 2020-2100, 2020-2200, and 2020-2300. The experts provided their quantiles absent the effects of climate damages to preclude double counting in the calculation of damages in the full SC-CO_2 calculation.

Continuous cumulative distribution functions (CDF) were generated for each expert by fitting each expert's five quantiles for each year elicited, in log GDP per capita, with a Johnson S_U distribution [120]. The CDFs for each expert were then combined by performance- weighting the experts based on their performance on the initial 11 calibration questions to yield a set of combined elicited values of OECD GDP per capita for each elicited year and quantile.

The results from the EGS were next used to reweight the probability of occurrence of each of the 2,000 runs from the MSW methodology, thereby preserving the country-level information from the econometric model while incorporating information from the experts. Importantly, the reweighting process did not modify the underlying projections from MSW; it modified the

probability of sampling a given set of trajectories to incorporate information on OECD growth drawn from the experts in the EGS.

The reweighting involves a two-step process. In the first step, target quantiles were generated for the years 2030, 2050, 2100, 2200, and 2300 by calculating weighted averages of CDFs from the raw MSW data and the combined CDFs from the experts. To generate the target quantiles for reweighting for each of the five target years, the relative weighting of the expert information versus the MSW model results begins at zero in 2030 and is increased linearly over time to reach 100% in 2200 and subsequent years, reflecting the expectation that the strength of the statistical model is greatest in the near- and medium-term. The near-term weighting is therefore more reliant on historically based statistical evidence; the long-term weighting is governed more by expert judgment, consistent with Morgan and Keith.[69]

In the second step, iterative proportional fitting [121] is used to impose the target quantiles for OECD growth on the 2,000 trajectories of the frontier from MSW for each of the five benchmark years, reassigning probabilities for each trajectory to match the target quantiles. If the algorithm converges (which it did in these cases), it converges to a distribution which is minimally informative with respect to the initial MSW distribution among all distributions satisfying the experts' quantile constraints.

Emissions

To quantify uncertainty in future emissions over the long time-horizon required for SC-CO_2 calculations, Resources for the Future conducted its Future Emissions Survey (FES) in 2021. Ten experts, based at universities, non-profit research institutions, and multilateral international organizations were surveyed individually over videoconference in roughly 2-hour interviews. Each expert had been nominated by their peers and/or members of the Scientific Advisory Board for RFF's Social Cost of Carbon Initiative and had expertise in long-term projections of the energy-economic system under a substantial range of climate mitigation scenarios.

The RFF Future Emissions Survey, similar to the Economic Growth Survey, employed the Classical Model of expert judgment. Experts first quantified their uncertainty for initial questions for which the answers were provided to them to familiarize them with the methodology and alert them to

potential biases. They next provided their quantiles of uncertainty (5th, 50th, and 95th percentiles) for a set of calibration questions, to allow for their performance-based weights to be used for combining the experts' distributions. Experts then provided quantiles of emissions for four years (2050, 2100, 2200, 2300) for four different categories of emissions: (1) fossil fuel and process-related CO_2 emissions; (2) changes in natural CO_2 stocks and negative-emissions technologies; (3) CH_4; and (4) N_2O, for five benchmark years: 2050, 2100, 2150, 2200, and 2300. The experts were explicitly asked to account for their own expectations on future policy and its efficacy, in addition to their expectations on all other relevant developments such as the pace of technology improvements and deployment of energy technologies, in the uncertain distributions they provided.

Experts were required to provide minimum, maximum, as well as 5, 50th, and 95th percentiles for each year for each of the above categories. At their discretion, experts were permitted to provide additional shape to their distributions by specifying emissions values for additional quantiles. Distributions were generated for each expert for each elicited year by piecewise linearly connecting their provided quantiles.

For direct emissions of CO_2 (category 1), experts were additionally required to condition their emissions quantiles on economic growth. This conditioning was accomplished by the experts specifying emissions quantiles for each elicited year for five different global average GDP per capita values spanning a very low to very high very high economic growth future. Experts were permitted, but not required, to condition the other emissions categories on economic growth. The expert distributions were combined according to their performance weights, resulting in a combined set of emissions distributions for each elicited year, conditioned on economic growth.

The process to generate the full set of internally consistent joint distributions across economic growth, population, and emissions was as follows: 10,000 independent samples of GDP/capita and population trajectories were sampled. Each sample was multiplied at the country level to calculate country-level GDP, and the results were summed across countries, then divided by the sum of the country-level population projections to generate globally averaged projections of GDP/capita for that sample.

Next, the following process was carried out 10,000 times to generate a joint distribution with net CO_2 emissions: (1) A value *q* was sampled on the interval [0,1]; (2) a sample was drawn from the 10,000 joint pathways GDP/capita and population; (3) based upon the pathway of GDP/capita drawn, interpolated emissions distributions for category 1 emissions were generated at 5 year intervals based upon the relevant emissions quantiles bounding the growth pathway for that year; (4) an emissions trajectory for category 1 was constructed by linearly interpolating in time the qth quantile of the resulting emissions distributions; and (5) an analogous process was carried out for natural carbon stocks and negative emissions (category 2) using an independent sample of *q*, and the results of the category 1 and category 2 trajectories were summed, thereby including the possibility of net negative emissions. For each of the 10,000 resulting net emissions trajectories, a cubic spline was used to interpolate between 2020 emissions and 2050 (the first year for which the experts provided emissions quantiles) based on the slope of the global emissions trajectories over the 2010-2020 period and the emissions trajectory post 2050. A cubic spline was additionally used to interpolate trajectories between the additional years for which experts provided quantiles.

Each expert's elicitation concluded with a validation step, in which the above sampling was performed for the expert in real time, based solely on that expert's provided quantiles. This step showed the resulting distributions of future emissions when sampled with the MSW economic growth and Raftery and Ševčíková population datasets. It additionally showed the resulting climate outputs to the year 2300, including atmospheric concentrations and globally averaged temperatures, by using that expert's sampled emissions distributions as inputs to the FaIR climate model.[71] Experts used this information to assure that the resulting distributions based upon the quantiles they had provided were in line with their expectations when sampled with the specific population and economic growth datasets with which they would be sampled. Experts were permitted to revise their quantiles if desired.

Discussion

Novel features of the RFF-SPs allow the GIVE model to estimate the SC-CO_2 in ways that specifically address the needs of policy analysis.

The native inclusion of uncertainty around future expectations of policy, as determined by the individual experts in the future emissions survey, is a novel and critical element for estimating the SC-CO_2 for use in regulatory analysis; the benefit-cost analysis approach employed evaluates regulations as incremental against the broader backdrop of other policies and conditions. The expert-specific inclusion of policy, without enforcing a specific policy pathway, is an essential feature of the emissions survey design, in that it treats future policy as a fundamental element of the overall uncertainty quantified by the experts.

A consequence of the survey's approach to policy inclusion is that recent developments in climate policy that have occurred since the survey, including the latest recent long-term, net-zero pledges made by a number of countries around COP26, are represented within the distributions provided by each of the experts only to the extent that contributing experts anticipated these future developments and incorporated them into their assessed probabilities of future emissions policies. As such, each expert provided probabilities (as quantified by their distributions) over the development of future pledges and the likelihood of their relative success.

The survey design and its interface allowed each expert to represent their view of the uncertain emissions future, inclusive of uncertainty over the ambition of future climate policy, with flexible control over the shape and temporal evolution of their emissions distributions. The ability to condition future emissions distributions on economic growth allowed for further tailoring and representation of experts' narratives. For example, a common narrative associated with high economic growth scenarios and reflected in the experts' emissions distributions was that high economic growth would lead to significantly increased emissions growth in the near-term, followed by a more rapid pace of decarbonization in the medium- and long-term due to a more rapid development and deployment of clean energy technologies.

The multi-century timescale required of projections to support SC-CO_2 calculations presents inherent research challenges. The RFF-SPs, through their novel combination of statistical and expert-based approaches, were designed specifically to address these challenges in a manner that quantifies the uncertainty. The resulting combined distributions for each of the variables are very wide in 2300, with global population ranging from 2.8 billion to 21 billion people, cumulative GDP per capita growth ranging from 0.17% to 2.7% per annum, and global annual CO_2 emissions

ranging from -14 to 36 gigatons (5th to 95th percentile ranges). This reflects the considerable uncertainty expected by the experts over such an extended time horizon. Ho et al.[37] also quantified expert uncertainty around future emissions to 2100 using a ratio-based approach for comparison with emissions from the SSPs and other methods. The RFF-SPs capture the interrelationships between future emissions and on economic growth, greater flexibility in inclusion of future policy developments, and multi-century timescale of the future emissions survey provide distinct additional features for policy analysis beyond the Ho et al. 2019 approach. Alternate, non-expert based approaches to generating very long-run socioeconomic projections have typically relied on extending existing, non-probabilistic scenarios mechanically or through other means, well beyond their original time horizon, often in ways distinct from the methods underlying the original projections.[63,70–72]

The probabilistic nature of the RFF-SPs is essential to the GIVE model's ability to propagate compounding uncertainty throughout each step of the estimation process and allows for the calibration of the economic discounting parameters and associated incorporation of risk valuation. The use of socioeconomic scenarios is an alternate approach that is often used in integrated assessment models to explore the sensitivity of SC-CO_2 results to specific storylines. Scenarios typically do not come with associated probabilities, precluding the proper calibration of the discounting parameters for them within the GIVE framework,[31] thereby complicating the direct comparison to results derived using probabilistic socioeconomic projections. Sensitivity analysis of the SC-CO_2 estimates was conducted using representative deterministic scenarios (SSPs 1,2,3, and 5) in Rennert et al.[3] using three of the four elements of the GIVE model (RFF-SP's, FaIR climate model, and discounting approach). That analysis, which by necessity used constant discounting for the SSPs rather than the stochastic discounting enabled with probabilistic projections, showed very different SC-CO_2 estimates across the range of SSPs, varying by a factor of 4 to 6 for 3% and 2% discount rates, respectively. This range of SC-CO_2 estimates across SSPs is very wide, and without probabilities associated with them, it is not possible to calculate an expected value across them to yield a meaningful quantitative central estimate or distribution for use in policy evaluation.

Results incorporating expert input are inherently somewhat sensitive to the specific cohort of experts sampled. The experts elicited for the future emissions survey and the economic growth survey, as well as the expert reviewers for the population model, are thought leaders in their respective fields individually, and the cohort as a whole was diverse across ethnicity, age, geography, and perspective. The results shown for the quantiles of individual experts for economic growth and future emissions (Figs. 4 and 7 of Rennert et al.[3]) are quite varied, suggesting that both surveys were successful at sampling a diverse set of perspectives. The demographers that formally reviewed the population model similarly provided a varied range of perspectives, from which a rough consensus for parameter distributions was drawn. The group consisted of prominent members of almost all the major population research groups (UN Population Division, IIASA, Max Planck Institute for Demographic Research, University of Washington, Uncertain Population of Europe project, etc.). The classical model of expert judgment[115,116] employed in the economic growth and future emissions surveys prioritizes validation with regard to uncertainty quantification, via the calibration questions.

SI.2 FaIR Climate Model

As discussed in the *Methods*, FaIR is designed to replicate the equilibrium and impulse-response behaviours found in more sophisticated Earth system models. These features are not found in the previous climate models used for SC-CO_2 calculations, which lack carbon cycle feedbacks and have been shown to respond too slowly to changes in radiative forcing. For these reasons and more, the NASEM report highlighted FaIR as an exemplary model satisfying the recommended criteria that climate models informing SC-CO_2 estimates should (i) represent current scientific understanding on the relationship between CO_2 emissions, atmospheric CO_2 concentrations, temperature responses, and the corresponding uncertainty levels (ii) capture the key dynamics and impulse-response behaviours observed in more sophisticated Earth system models, (iii) be relatively simple and transparent, and (iv) account for non-CO_2 emissions and radiative forcings. The recent IPCC AR6 Reports also used version 1.6.2 of the FaIR model to carry out a number of analyses. That original version of the model is stored online at https://github.com/OMS-NetZero/FAIR. In particular, Working Group 1 relied on FaIR to help assess global-mean surface temperature responses, Global Temperature change Potential metrics, and their associated

uncertainty levels across five illustrative SSP-RCP scenarios[75] while Working Group 3 used FaIR to quantify the climate responses across a much wider range of potential mitigation and emission pathways.[122]

We follow the IPCC's approach for quantifying climate projection uncertainty in FaIR by randomly sampling the same set of constrained model parameters used throughout the analyses presented in the AR6 report.[75] To generate these parameter samples, IPCC authors first used FaIR to produce a one-million-member ensemble of historic model runs from 1750 to 2019. These model runs sampled parametric uncertainties from distributions informed by past observations as well as results from the recent Coupled Model Intercomparison Project 6 exercise. The uncertain FaIR parameters include the effective heat capacities of the surface and deep ocean layers, deep ocean heat uptake efficacy, the integrated airborne fraction of CO_2 over the pre-industrial period, the strength of different forcing agents, a climate feedback term, and a number of other key model parameters described more fully in Supplementary Information Table 3.

Each historic model run in the one-million-member ensemble was then evaluated based on its ability to meet three observational constraints:

1. Produce a root-mean-square error no larger than 0.135°C when compared to the assessed AR6 1850-2020 surface temperature time series
2. Produce 1971-2018 ocean heat uptake values that fall within the range of 329-463 ZJ
3. Produce 2014 atmospheric CO_2 concentrations that are within the range of 397.1 ± 0.4ppm

In addition, the FaIR ensemble members were also constrained against 1% per year CO_2 increase perturbation modelling experiments to account for the fraction of anthropogenic CO_2 emissions that remain in the atmosphere due to ocean and land carbon sink dynamics and climate-carbon cycle feedbacks. Specifically, FaIR ensemble members had to produce an airborne fraction value falling between $53 \pm 6\%$.

The IPCC AR6 calibration process yielded 2,237 parameter combinations for FaIR that passed all four modelling constraints out of the original one million possibilities (Supplementary

Information Table 3). We run FAIR by randomly sampling these 2,237 parameters sets with replacement and also randomly sample CO_2, CH_4, and N_2O emissions time-series from the RFF-SP scenarios. We represent other greenhouse gases and short-lived climate forcers using the SSP2-4.5 scenario, the scenario that most closely matches the median RFF-SP emissions trajectories.

SI.3 Recent Advances in SC-CO_2 Estimation

The integrated assessment modelling and SC-CO_2 literature has seen important contributions in recent years. We here briefly review some particularly important papers and highlight how they relate to the current study. We focus on modelling innovations, noting that some of the papers we describe do not estimate an SC-CO_2.

Several studies have used existing integrated assessment models (typically DICE) and replaced the damage function with a specification that is based on reduced form empirical estimates relating temperature to GDP growth based on historical data. The first paper to do this was Moore and Diaz[22], who introduce growth-rate damage estimated by Dell, Jones, and Olken[123] into the TFP growth rate and the capital depreciation damage parameters of the DICE model. Glaneman, Willner, and Levermann[24] introduce a damage function estimated in Burke, Hsiang, and Miguel (BHM)[6] into DICE to perform a cost-benefit test of the Paris Climate agreement. Damage function parametric uncertainty is quantified, and sensitivity analyses are performed for alternative values of the equilibrium climate sensitivity and for different socioeconomic scenarios (using DICE and three SSPs) and preference specifications. Gazzotti et al.[26] construct a modified version of DICE that uses the BHM damage function but focuses on regional disaggregation to estimate non-cooperative equilibrium policy and its implications for inequality of outcomes; uncertainty is not explicitly quantified but is treated using sensitivity analyses encompassing multiple socioeconomic scenarios and damage functions. Hänsel et al.[25] update DICE using the damage function from Howard and Sterner[33], but treatment of uncertainty is limited to consideration of the range discounting parameters from the survey of Drupp et al.[52]

Ricke et al.[23] estimates country-level SC-CO_2 values based on the BHM damage estimates. Socioeconomic uncertainty is treated via a sensitivity analysis using different SSP scenarios,

climate system uncertainty by using output from an ensemble of climate models, and damage function uncertainty is quantified via parametric uncertainty based on BHM.

Bressler[27] augments DICE to calculate the mortality cost of carbon, finding large values from reducing CO_2 emissions due to lower mortality rates valued using a global uniform VSL, but relies primarily on DICE's outdated formulation of climate and socioeconomic features. Treatment of uncertainty is limited to the magnitude of the response of mortality to temperature.

SI.4 Parameterization of the GIVE Model and Uncertainty

Region	Estimated Coefficient (β_i^M)	Standard Error
Latin America	0.179%	0.054%
Europe	0.110%	0.028%
Sub-Saharan Africa	0.238%	0.076%
South Asia	0.391%	0.467%
Eastern Europe	0.455%	0.073%
USA	0.464%	0.204%
Southeast Asia	0.431%	0.182%
Australia	0.452%	0.133%
China & East Asia	0.535%	0.068%
Middle East & North Africa	1.103%	0.829%

Supplementary Table 1: Regional temperature-related mortality coefficients. The left column shows the regions used in Cromar et al. (2022), the source of the GIVE model's temperature-related mortality damage function. The middle column shows the estimated coefficient between temperature rise (degrees Celsius) and all-cause mortality risk for a given region. The right column shows the standard errors associated with these estimates. More precise values are available in the replication code repository.

Region	Estimated Coefficient (β_i^E)
Brazil	0.077%
Canada	-0.006%
China	0.050%
Europe	0.008%
India	0.057%
Middle East & North Africa	0.128%
Other Asia	0.110%
Other Latin America	0.049%
Pacific OECD	0.013%
Russia	-0.046%
Sub-Saharan Africa	0.119%
USA	0.038%

Supplementary Table 2: Regional energy expenditure damages coefficients. The left column shows the regions used in Clarke et al. (2018), the source of the GIVE model's energy expenditure damage function. The right column shows the estimated coefficient between temperature rise (degrees Celsius) and change in energy expenditures as a portion of GDP for a given region. More precise values are available in the replication code repository.

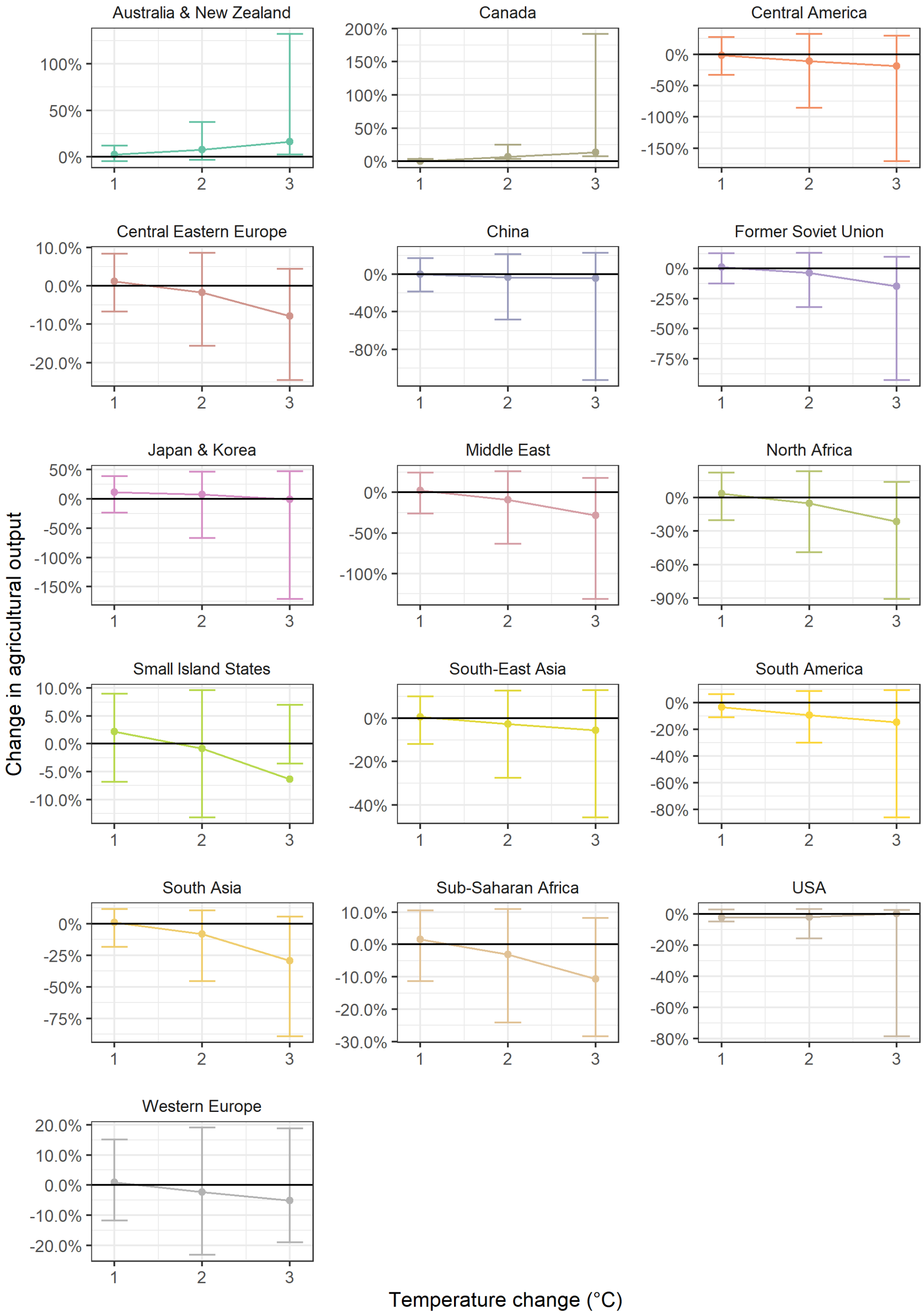

Supplementary Figure 1: Regional agricultural damages coefficients. Each panel in the figure shows the change in agricultural output for one of the regions used in Moore et al.,[18] the source of the GIVE model's agricultural damage function. Values are presented as a percentage of initial agricultural output for each region. Each panel contains nine vertices representing the low (bottom error bar), middle (point), and high (top error bar) welfare scenarios for each of three different temperature increases (1, 2, and 3 degrees Celsius). Data are available in the replication code repository.

Uncertain Model Parameters	Units	Parameter Quantiles		
		5%	50%	95%
Efficacy of deep ocean heat uptake	—	0.6137	1.293	2.000
Ocean heat capacity (mixed layer)	$W\ m^{-2}\ yr^{-1}\ °C^{-1}$	6.031	8.324	10.12
Ocean heat capacity (deep ocean)	$W\ m^{-2}\ yr^{-1}\ °C^{-1}$	58.51	122.5	258.5
Ocean heat exchange coefficient between mixed and deep layers	$W\ m^{-2}\ °C^{-1}$	0.4659	0.6552	0.8940
Climate feedback parameter	$W\ m^{-2}\ °C^{-1}$	0.8038	1.367	1.947
Effective radiative forcing from a doubling of atmospheric CO_2	$W\ m^{-2}$	3.563	4.037	4.498
Pre-industrial atmospheric CO_2 concentrations	Ppm	272.1	276.9	281.5
Pre-industrial 100-year time-integrated airborne fraction of CO_2	Yr	28.42	32.71	37.90
Sensitivity of 100-year time-integrated airborne fraction to atmospheric carbon	$yr\ GtC^{-1}$	6.581×10^{-3}	0.02495	0.04341
Sensitivity of 100-year time-integrated airborne fraction to temperature	$yr\ °C^{-1}$	0.1530	2.143	4.252
Aerosol radiative efficiency coefficient (SOx)	$Wm^{-2}\ (Mt\ yr^{-1})^{-1}$	-9.938×10^{-3}	-6.536×10^{-3}	-3.407×10^{-3}
Aerosol radiative efficiency coefficient (NH_3)	$Wm^{-2}\ (Mt\ yr^{-1})^{-1}$	-2.858×10^{-3}	-1.981×10^{-3}	-1.067×10^{-3}
Aerosol radiative efficiency coefficient (OC)	$Wm^{-2}\ (Mt\ yr^{-1})^{-1}$	-8.609×10^{-3}	-4.930×10^{-3}	-1.243×10^{-3}
Aerosol radiative efficiency coefficient (BC)	$Wm^{-2}\ (Mt\ yr^{-1})^{-1}$	0.01507	0.04242	0.06968
Aerosol indirect effect scaling factor	—	0.3902	3.414	2.534×10^{4}
Aerosol indirect effect sensitivity to SOx emissions	—	3.256	390.8	5.259×10^{6}
Aerosol indirect effect sensitivity to BC and OC emissions	—	1.958	224.3	1.341×10^{6}
Temperature feedback on O3 effective radiative forcing	$W\ m^{-2}\ °C^{-1}$	-0.05649	-0.03725	-0.01766
O_3 radiative efficiency coefficient (CH_4)	$W\ m^{-2}\ ppb^{-1}$	1.141×10^{-4}	1.748×10^{-4}	2.376×10^{-4}
O_3 radiative efficiency coefficient (N_2O)	$W\ m^{-2}\ ppb^{-1}$	2.561×10^{-4}	7.104×10^{-4}	1.193×10^{-4}
O_3 radiative efficiency coefficient (CO)	$W\ m^{-2}\ (Mt\ yr^{-1})^{-1}$	2.559×10^{-5}	1.566×10^{-4}	2.877×10^{-4}
O_3 radiative efficiency coefficient (NMVOCs)	$W\ m^{-2}\ (Mt\ yr^{-1})^{-1}$	8.311×10^{-7}	3.336×10^{-4}	6.701×10^{-4}
O_3 radiative efficiency coefficient (NOx)	$Wm^{-2}\ (MtN\ yr^{-1})^{-1}$	2.731×10^{-3}	6.061×10^{-3}	9.226×10^{-3}
O_3 radiative efficiency coefficient (ozone-depleting substances)	$W\ m^{-2}\ ppt^{-1}$	-2.290×10^{-4}	-1.177×10^{-4}	-2.030×10^{-6}
Time series of effective radiative forcing from solar irradiance*	$W\ m^{-2}$	—	—	—
Effective radiative forcing scaling term (CH_4)	—	0.6846	0.8639	1.033
Effective radiative forcing scaling term (N_2O)	—	0.9207	1.072	1.216
Effective radiative forcing scaling term (black carbon on snow)	—	-0.02466	1.016	2.250
Effective radiative forcing scaling term (land use surface albedo change)	—	0.4963	0.9969	1.494
Effective radiative forcing scaling term (volcanic)	—	0.7385	0.9903	1.231
Effective radiative forcing scaling term (stratospheric H_2O from CH_4 oxidation)	—	-0.03505	1.006	2.022
Effective radiative forcing scaling term (CF_4)	—	0.8140	1.001	1.188
Effective radiative forcing scaling term (C_2F_6)	—	0.8140	1.001	1.188
Effective radiative forcing scaling term (C_6F_{14})	—	0.8140	1.001	1.188
Effective radiative forcing scaling term (HFC-23)	—	0.8140	1.001	1.188
Effective radiative forcing scaling term (HFC-32)	—	0.8140	1.001	1.188
Effective radiative forcing scaling term (HFC43-10)	—	0.8140	1.001	1.188
Effective radiative forcing scaling term (HFC-125)	—	0.8140	1.001	1.188
Effective radiative forcing scaling term (HFC-134a)	—	0.8140	1.001	1.188
Effective radiative forcing scaling term (HFC-143a)	—	0.8140	1.001	1.188
Effective radiative forcing scaling term (HFC-227ea)	—	0.8140	1.001	1.188
Effective radiative forcing scaling term (HFC-245fa)	—	0.8140	1.001	1.188
Effective radiative forcing scaling term (SF_6)	—	0.8140	1.001	1.188
Effective radiative forcing scaling term (CFC-11)	—	0.9198	1.131	1.343
Effective radiative forcing scaling term (CFC-12)	—	0.9116	1.121	1.331
Effective radiative forcing scaling term (CFC-113)	—	0.8140	1.001	1.188
Effective radiative forcing scaling term (CFC-114)	—	0.8140	1.001	1.188
Effective radiative forcing scaling term (CFC-115)	—	0.8140	1.001	1.188
Effective radiative forcing scaling term (CCl_4)	—	0.8140	1.001	1.188
Effective radiative forcing scaling term (Methyl chloroform)	—	0.8140	1.001	1.188
Effective radiative forcing scaling term (HCFC-22)	—	0.8140	1.001	1.188
Effective radiative forcing scaling term (HCFC-141b)	—	0.8140	1.001	1.188
Effective radiative forcing scaling term (HCFC-142b)	—	0.8140	1.001	1.188
Effective radiative forcing scaling term (Halon 1211)	—	0.8140	1.001	1.188
Effective radiative forcing scaling term (Halon 1202)	—	0.8140	1.001	1.188
Effective radiative forcing scaling term (Halon 1301)	—	0.8140	1.001	1.188
Effective radiative forcing scaling term (Halon 2402)	—	0.8140	1.001	1.188
Effective radiative forcing scaling term (CH_3Br)	—	0.8140	1.001	1.188
Effective radiative forcing scaling term (CH_3Cl)	—	0.8140	1.001	1.188

Supplementary Table 3: Uncertain parameters and their quantile values for the FAIR v1.6.2 climate model. Columns under the "Parameter Quantiles" heading show the 5th, 50th, and 95th quantile values for the calibrated parameter distributions produced as part of chapter 7 of the IPCC AR6 report[75]. Quantiles correspond to the 2,237-member set of IPCC parameters. We sample uncertain effective radiative forcing time series for solar irradiance from 1750 to 210. After 2110, solar forcing follows the SSP2-4.5 scenario. SC-CO_2 results calculated using 10,000 samples from these parameters with replacement.

Uncertain Model Parameters	Units	Parameter Quantiles		
		5%	50%	95%
Global ocean-averaged thermal expansion coefficient	kg m^{-3} °C^{-1}	0.1453	0.1885	0.2316
Sensitivity of Greenland ice sheet equilibrium volume to temperature changes	m SLE °C^{-1}	-3.249	-1.579	-0.7212
Greenland ice sheet equilibrium volume for 0 °C global temperature anomaly	m SLE	7.550	8.030	8.702
Temperature sensitivity of Greenland ice sheet exponential decay rate	yr^{-1} °C^{-1}	1.804×10^{-4}	4.654×10^{-4}	9.218×10^{-4}
Exponential decay rate for 0 °C global temperature anomaly (Greenland)	yr^{-1}.	3.415×10^{-6}	4.778×10^{-5}	2.932×10^{-4}
Initial glaciers and small ice caps mass-balance sensitivity to global temperature	m °C^{-1} yr^{-1}	7.435×10^{-4}	8.705×10^{-4}	1.059×10^{-3}
Exponential parameter for glaciers and small ice caps area to volume scaling	—	0.5741	0.7761	0.9796
Slope of undisturbed Antarctic ice sheet bed before loading	—	5.457×10^{-4}	6.434×10^{-4}	7.220×10^{-4}
Profile parameter for parabolic ice sheet surface (Antarctic)	m$^{1/2}$	7.566	10.65	13.31
Height of Antarctic ice sheet runoff line above which precipitation accumulates as snow for 0 °C local temperatures	m	807.4	1330	2048
Proportionality constant for the dependency of runoff line height on local Antarctic surface temperature	(m/°C)	60.50	105.8	137.5
Annual precipitation for 0 °C local Antarctic temperature	m ice equivalent yr^{-1}	0.2274	0.8173	1.408
Coefficient for precipitation exponential dependency on Antarctic temperature	—	0.03802	0.06673	0.08287
Proportionality constant relating Antarctic ice sheet runoff decrease with height to precipitation	—	3.596×10^{-3}	8.579×10^{-3}	0.01421
Proportionality constant for ice flow at the Antarctic ice sheet grounding line	m yr^{-1}	0.6909	1.260	1.737
Power for the relation of Antarctic ice flow speed to water depth	—	1.209	2.891	4.107
Undisturbed Antarctic ice sheet bed height at the continent centre	m	744.6	778.6	815.4
Partition parameter for ocean subsurface temperature effect on Antarctic ice flux	—	0.01451	0.1373	0.5783
Trigger temperature for Antarctic ice sheet fast dynamics disintegration to begin	°C	-16.22	-15.67	-14.83
Antarctic ice sheet fast dynamics disintegration rate	m yr^{-1}	5.295×10^{-3}	9.885×10^{-3}	0.01688
Antarctic Ocean temperature sensitivity to global surface temperature	°C / °C	0.01350	0.1360	0.6614
Antarctic ocean temperature for 0 °C global surface temperature anomaly	°C	0.09736	0.9183	1.872
Initial sea-level rise due to thermal expansion	m SLE	-0.04357	-2.824×10^{-5}	0.04351
Initial volume of Greenland ice sheet	m SLE	7.178	7.356	7.539
Initial total volume of glaciers and small ice caps	m SLE	0.3204	0.4159	0.5182
Initial cumulative sea-level rise from glaciers and small ice caps	m	-0.04556	0.01897	0.07362
Initial sea-level rise from Antarctic ice sheet	m	-0.04226	4.009×10^{-3}	0.05074
AR(1) standard deviation (glaciers and small ice caps)	m	1.738×10^{-5}	1.866×10^{-4}	5.928×10^{-4}
AR(1) standard deviation (Greenland ice sheet)	m	1.983×10^{-4}	2.385×10^{-4}	2.897×10^{-4}
AR(1) standard deviation (Antarctic ice sheet)	m	1.760×10^{-4}	3.804×10^{-4}	6.088×10^{-4}
AR(1) standard deviation (global mean sea-level)	m	4.642×10^{-4}	1.791×10^{-3}	2.942×10^{-3}
AR(1) Autocorrelation (glaciers and small ice caps)	—	-0.8125	0.04002	0.8436
AR(1) Autocorrelation (Greenland ice sheet)	—	0.8757	0.9520	0.9859
AR(1) Autocorrelation (Antarctic ice sheet)	—	0.8139	0.9445	0.9857
AR(1) Autocorrelation (global mean sea-level)	—	0.06766	0.9892	0.9990

Supplementary Table 4: Uncertain parameters and their quantile values for the BRICK sea-level model. Columns under the "Parameter Quantiles" heading show the 5th, 50th, and 95th quantile values for the calibrated parameter distributions.

Supplementary information references

110. Aspinall, W. A route to more tractable expert advice. *Nature* **463**, 294–295 (2010).

111. Bamber, J. L. & Aspinall, W. P. Expert Judgement Assessment of Future Sea Level Rise from the Ice Sheets, An. *Nat. Clim. Change* **3**, 424–427 (2013).

112. Bamber, J. L., Oppenheimer, M., Kopp, R. E., Aspinall, W. P. & Cooke, R. M. Ice sheet contributions to future sea-level rise from structured expert judgment. *Proc. Natl. Acad. Sci.* 201817205 (2019).

113. Azose, J. J. & Raftery, A. E. Bayesian Probabilistic Projection of International Migration. *Demography* **52**, 1627–1650 (2015).

114. Azose, J. J., Ševčíková, H. & Raftery, A. E. Probabilistic population projections with migration uncertainty. *Proc. Natl. Acad. Sci. U. S. A.* **113**, 6460–6465 (2016).

115. Cooke, R. M. *Experts in Uncertainty: Opinion and Subjective Probability in Science*. (Oxford University Press, 1991).

116. Cooke, R. M. Uncertainty analysis comes to integrated assessment models for climate change…and conversely. *Clim. Change* **117**, 467–479 (2013).

117. Colson, A. R. & Cooke, R. M. Cross validation for the classical model of structured expert judgment. *Reliab. Eng. Syst. Saf.* **163**, 109–120 (2017).

118. Colson, A. R. & Cooke, R. M. Expert Elicitation: Using the Classical Model to Validate Experts' Judgments. *Rev. Environ. Econ. Policy* **12**, 113–132 (2018).

119. Cooke, R. M., Marti, D. & Mazzuchi, T. Expert forecasting with and without uncertainty quantification and weighting: What do the data say? *Int. J. Forecast.* **37**, 378–387 (2021).

120. Johnson, N. L. Systems of Frequency Curves Generated by Methods of Translation. *Biometrika* **36**, 149–176 (1949).

121. Csiszar, I. I-Divergence Geometry of Probability Distributions and Minimization Problems. *Ann. Probab.* **3**, 146–158 (1975).

122. IPCC, 2021: Annex III: Tables of historical and projected well-mixed greenhouse gas mixing ratios and effective radiative forcing of all climate forcers. in *Climate Change 2021: The Physical Science Basis. Contribution of Working Group I to the Sixth Assessment Report of the Intergovernmental Panel on Climate Change* (eds. Dentener, F. J., Hall, B. & Smith, C.) (2021).

123. Dell, M., Jones, B. F. & Olken, B. A. Temperature Shocks and Economic Growth: Evidence from the Last Half Century. *Am. Econ. J. Macroecon.* **4**, 66–95 (2012).
